# Ovarian responses and FSH profiles at superovulation with a single epidural administration of gonadotropin in the Thai-Holstein crossbreed

**DOI:** 10.1590/1984-3143-AR2021-0053

**Published:** 2021-11-04

**Authors:** Rujira Chumchai, Thanaporn Ratsiri, Ruthaiporn Ratchamak, Thevin Vongpralub, Wuttigrai Boonkum, Vibuntita Chankitisakul

**Affiliations:** 1 Department of Animal Science, Faculty of Agriculture, Khon Kaen University, Khon Kaen, Thailand; 2 The Research and Development Network Center of Animal Breeding and Omics, Khon Kaen University, Khon Kaen, Thailand

**Keywords:** Embryo transfer, superstimulation, dairy cows, ovarian cysts

## Abstract

The conventional method of ovarian superstimulation requires multiple injections of gonadotropins which is time-consuming and may be stressful for the cows. This study was designed to determine whether a single epidural injection of FSH (EI group) would induce the superovulatory response in the Thai-Holstein crossbreed and evaluate FSH plasma hormone concentrations. Eight cows (replication = 3; *n*=24) were assigned to one of 2 treatments in switch back design. Control group (*n*=12): cows were received 400 mg FSH twice daily by intramuscularly for 4 days (80, 80, 60, 60, 40, 40, 20 and 20 mg), EI group (*n*=12): cows were received 400 mg FSH by single epidural injection. Data were collected in term of ovarian follicle responses, superovulatory responses, ova/embryo collection. FSH concentrations were examined using ELISA. The total follicular responses during oestrus were not different between treatments; however, the large follicles were less frequent (*P* < 0.01) while the medium follicle sizes were higher (*P* < 0.05) in the EI group. The plasma concentration of FSH in EI was dramatically increased within 2 hours before decreasing sharply thereafter (*P* < 0.01) and did not remain above baseline after 10 hours of administration. The embryo quality was better in the control than the EI groups (*P* < 0.05). Interestingly, the number of ovulation cysts in the EI group was 50%. The ovarian responses and embryo quality in the cows with cysts were worse compared with the non-cyst groups (*P* < 0.05). In conclusion, alternative protocols decreased the superovulatory response and increased poor embryo quality in Thai-Holstein crossbred. Also, the incidence of ovarian follicular cysts is higher in the EI group.

## Introduction

To increase the numbers of high genetic animals, superovulation and embryo transfer are effective tools which are widely used in many countries (Parker Gaddis et al., 2017). Follicle stimulating hormone (FSH), exogenous gonadotrophic hormone, is commonly used to stimulate follicular growth, and subsequently obtain a greater number of ovulations, leading to a high number of transferable embryos and a high probability of pregnancy after transfer (Mapletoft, 1986). However, multiple injections of FSH are required due to its short half-life, which is time-consuming and may also be stressful for the cows (Sugano and Shinogi, 1999; Chasombat et al., 2013). Many previous studies proposed solving this issue by administering FSH dissolved in various agents that provide a slow release, such as polyvinylpyrrolidone (PVP)(Yamamoto et al., 1994; Takedomi et al., 1995; Ratsiri et al., 2021) and aluminium hydroxide gel (AH-gel) in cattle (Kimura, 2016). However, those diluting agents make the solution viscous and difficult to prepare well with FSH. Besides, those protocols come with the cost of slow-release agents.

The reports of a single epidural injection (EI) of FSH for superstimulation response are limited. The first report was published in 2012 by Taşdemir (Taşdemir et al., 2012), who stated that EI plus an intramuscular injection (IM) of FSH could obtain acceptable results compared with conventional administration protocols; however, the transferable embryo rate in Anatolian Black cows (*Bos Taurus*) was unsatisfactory. A recent study reported that the EI of FSH could induce follicular growth followed by in vivo and in vitro embryo production in Japanese Black cow (Sakaguchi et al., 2018). It is speculated that epidural fats contribute to the slow movement of FSH into the peripheral circulation (Lee et al., 2003). However, successfully superstimulation in those beef cattle might fail in dairy cattle, as there is variation in epidural fat between two breeds. Similarly, many previous protocols could not be repeated in Holstein cows, which presumably have less subcutaneous adipose tissue (Bo et al., 1994). Therefore, the present research was designed to (1) compare the ovarian responses to superovulation between conventional administration and a single epidural administration of FSH, and (2) study those FSH plasma hormone concentrations during FSH administration in the Thai-Holstein crossbred.

## Methods

### Animal care

This study was reviewed and approved by the institution for animal care, based on the ethics of animal experimentation of the National Research Council of Thailand (Record No. IACUC-KKU-68/62, Reference No. 660201.2.11/81)

### Chemicals

All the chemicals used in this study were purchased from Sigma-Aldrich (St. Louis, MO, USA) unless otherwise stated.

### Animals, housing, and feeding

Thai-Holstein crossbreed donors (n=8) from the Agriculture Training and Experiment Station, Faculty of Agriculture, Khon Kaen University, were used as the donors in the experiment. All were non-lactating cows, aged from 3 to 6 years and had a good body condition score (BCS) ranging between 3 and 3.5 (1–5 scale). They were housed in comfortable housing, received fresh water, and were fed with optimized nutrition.

### Experimental design

**Figure 1:**
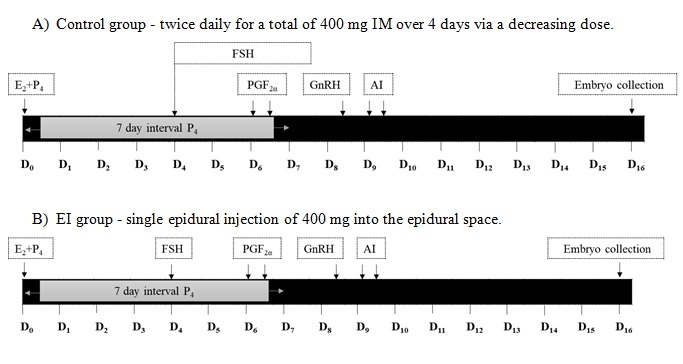
Treatment protocols. All cows received oestradiol plus progesterone and the intravaginal insertion of a 7 day progesterone device. FSH began on the fourth day after synchronisation. (A) In the control, the FSH treatment consisted of twice daily IM injection of a decreasing dose for 4 days; (B) The EI group was a single epidural injection of 400 mg into the epidural space between the 1^st^ and 2^nd^ coccygeal bone. All of the treated animals were administered prostaglandin F2α twice on Day 6 (12 hour intervals) IM. At twelve hours before AI, cows received GnRH IM. All cows were then artificially inseminated at 12 and 24 hour after GnRH administration with frozen-thawed semen. Seven days after insemination, embryos were collected.

All cows were randomly assigned to one of two treatments (control and EI groups) as described in Figure 1 to be superstimulated in a switch back design with 50-day rest period. In the control group, cows received 400 mg of FSH (Folltropin^®^-V; Bioniche Animal Health, Belleville, ON, Canada) diluted in 20 mL of saline given in a twice daily, decreasing dose (80, 80, 60, 60, 40, 40, 20, 20 mg, respectively), via IM injection over 4 days. In the EI group, cows received 400 mg of FSH dissolved in 5 mL of saline given into the epidural space between the 1^st^ and 2^nd^ coccygeal bone.

### Synchronization and superstimulation

On a random day of the oestrus cycle (Day 0), cows were synchronized with an intravaginal device impregnated with 1.56 g progesterone (Eazi-Breed CIDR-B^®^, Zoestis Animal Health, Kalamazoo, MI, USA) and 5 mg oestradiol-17β plus 50 mg progesterone (SRC Animal Health, Pak Chong, Nakhon Ratchasima, Thailand). On Day 4, all cows were superstimulated with FSH, according to the groups described above. All of the treated cows were administered 25 mg of prostaglandin F2α (Lutalyze^®^, Zoestis) IM twice on Day 6 (12 hour intervals) and CIDR-B was removed on the morning of Day 7. On the evening of Day 8, all cows received 2.5 mL of GnRH (Receptal^®^, MSD, Unterschleissheim, Germany) and were artificially inseminated with frozen-thawed semen 12 and 24 hours later. Seven days after insemination, embryos were collected (Figure 1).

### Ultrasonography evaluations

Ovarian follicular activity in terms of diameters and number of all larger follicles (≥5 mm in diameter) was examined by transrectal ultrasonography (HS-2000 ultrasound scanner; Honda Electronics Co., Toyohashi, Japan) 12 hours before artificial insemination. Seven days after insemination, the number of CLs and unovulated follicles (≥9 mm) were assessed before embryo collection. Animals with three or more CLs at the time of embryo collection were determined to have responded to the superovulatory treatment. The ovarian response of each animal to treatment was calculated by summing the anovulatory follicles to the CLs. The ovulation rate was calculated by dividing the number of CLs by the ovarian response. The ovarian cysts were characterized as follicular structures greater than 25 mm in diameter remaining at least 10 days by transrectal ultrasonography. Those cows were treated by administering 5 mL of GnRH.

### Blood sample collection and hormone analyses

To compare the FSH profile of two treatment groups, blood samples were collected from the jugular vein using an intravenous catheter for large animals and 6 mL vacutainer tubes containing heparin (VACUETTE®, Greiner Bio-One Co., Ltd., Austria). Blood collections were done at 0 hour just before the start of treatment, and 2, 4, 6, 8, 10, 24, 31, 48, 55, 72 and 79 hours after the initiation of the treatment (Hiraizumi et al., 2015). The samples were centrifuged at 350 x *g* for 20 min. The plasma was collected and stored at –20^o^C until measurements of hormone concentration.

Plasma concentrations of FSH were measured using competitive enzyme immunoassay kits (bovine FSH ELISA kit, My Biosource CO. LTD., Cat. no. MBS705623), with intra- and inter-assay CVs < 15% for both precision criteria. Analytical procedures were performed following the instructions provided by the manufacturer.

### Ova and embryo collection

Embryo recovery was performed on Day 16 using a standard non-surgical technique to flush the uterine horns. Uterine flushing was conducted with Dulbecco’s PBS supplemented with 1% fetal calf serum (Biological Industries, Beit Haemek, Israel), a silicone two-way Foley catheter, and a long flushing tube set. Firstly, epidural anesthesia was used to relax the rectal musculature using 5 mL of 2% lidocaine. To process uterine flushing, a Foley was setup with a dilator with a diameter of 7 mm, which was inserted into the cervix; the bulb of the Foley catheter sealed the head of the cervix. The balloon was inflated with approximately 12 mL of air, and the uterine horns were each flushed with 50 mL of DPBS until the total volume of flushing was 500 mL for each uterine horn (Chankitisakul et al., 2017). All flushing procedures were performed by the same experienced researcher under the inspection of a veterinarian. The unfertilized oocytes and embryos were visualized with a stereomicroscope (Olympus SZ40, Olympus, Japan). Recovered embryos were evaluated and graded according to their stage of development (i.e., ova, 2–8 cell, 8–16 cell, early morula, compacted morula, early blastocyst, blastocyst, and expanded blastocyst) and quality grade (A: excellent, B: good, C: fair, and D: poor) as the criteria of Lindner and Wright (Lindner and Wright 1983). Only embryos graded A and B were considered to be transferable embryos, and only embryos of quality graded A were determined as freezable embryos. Besides that, those were determined as unfertilized ova and degenerated embryos as our previous study (Chankitisakul et al., 2017).

### Statistical analysis

Data were analyzed using the SAS statistical software (1998). Data were first tested for normality and homogeneity of variance and then were analyzed by Proc ANOVA as a switch-back design. The study factors included treatment, period, and cows; the effects of treatment sequence and interaction among those factors were also considered. Treatment groups were compared for differences using Duncan’s New Multiple Range Test. The overall differences between treatment means were considered significant when *P* < 0.05. The full statistical model was as follows:


$$y_{ijkl}=\mu +\tau_{i}+\alpha_{j}+\delta_{k\left(j\right)}+\beta_{l}+\tau \beta_{il}+\tau \delta_{ik\left(j\right)}+\beta \delta_{lk\left(j\right)}+\varepsilon_{ijkl}$$ (1)

Where $y_{ijkl}=$ observation values of follicular responses, ovarian responses, and superovulatory response on treatment i (i = 1 to 2) at treatment sequence j (j = 1 to 2), cows k (k = 1 to 8), and period l (l = 1 to 3); μ= overall mean; $\tau_{i}=$ the effect of treatment i (i = 1 to 2); $\alpha_{j}=$ the effect of treatment sequence j (j = 1 to 2); $\delta_{k\left(j\right)}=$ the effect of cows k (k = 1 to 8) within treatment sequence j (j = 1 to 2); $\beta_{l}=$ the effect of period l (l = 1 to 3); $\tau \beta_{il}=$ the effect of interaction between treatment and period; $\tau \delta_{ik\left(j\right)}=$ the effect of interaction between treatment and cows within treatment sequence; $\beta \delta_{lk\left(j\right)}=$ the effect of interaction between period and cows within treatment sequence; $\varepsilon_{ijkl}=$ the effect of experimental error.

## Results

Treatment factor was significantly (*P* < 0.05) while main factors in term of cows (animal factor) and period, treatment sequence, interaction between treatment x period, treatment x cows, and period x cows were not significant (*P* > 0.05).

All cows (*n*=12 in each group) showed signs of oestrus and were inseminated artificially. However, five cows did not respond to the superstimulation (number of ovulations ≤ 2). The EI group showed less superstimulation compared to the control group (66.7 and 91.7%; *P* < 0.01).

The results of follicular response of two superstimulation protocols are presented in Table1. Total follicles between groups did not differ (19.5 ± 1.5 vs. 14.5 ± 1.5; *P* > 0.05). However, the number of large follicles in the control group was higher (17.1 ± 1.5 vs. 8.5 ± 1.5; *P* < 0.05) than in the EI groups, while there were more (3.7 ± 0.6 vs. 1.6 ± 0.5; *P* < 0.05) medium follicles in the EI group.

**Table 1:** Follicular responses between cows administered FSH by twice daily intramuscular injection or single epidural injection of 400 mg FSH in Thai-Holstein Friesian (Mean ±SE).

**Group**	**Animal (n)**	**Follicles** **5-8 mm(n) 1**	**Follicles** **>9 mm(n) ^1^**	**Total follicles(n) ^1^**
Control	12	1.6±0.5^b^	17.1±1.5a	19.5±1.5^a^
EI	12	3.7±0.6^a^	8.5±1.5b	14.5±1.5^a^
*P-*values		0.0325	0.0091	0.0694

^a,b^ For treatment effect, means within column are significantly different (*P* < 0.05) by Duncan’s multiple range test (DMRT). Control: FSH 400 mg continuous intramuscular injection twice a day for 4 days. EI: FSH 400 mg single epidural injection. ^1^ number of follicle 12 hours before first artificial insemination. SE is standard error of the sample mean calculated from $\frac{SD}{\sqrt{n}}$ when SD is standard deviation of sample size and $\sqrt{n}$ is square root of sample size.

**Figure 2:**
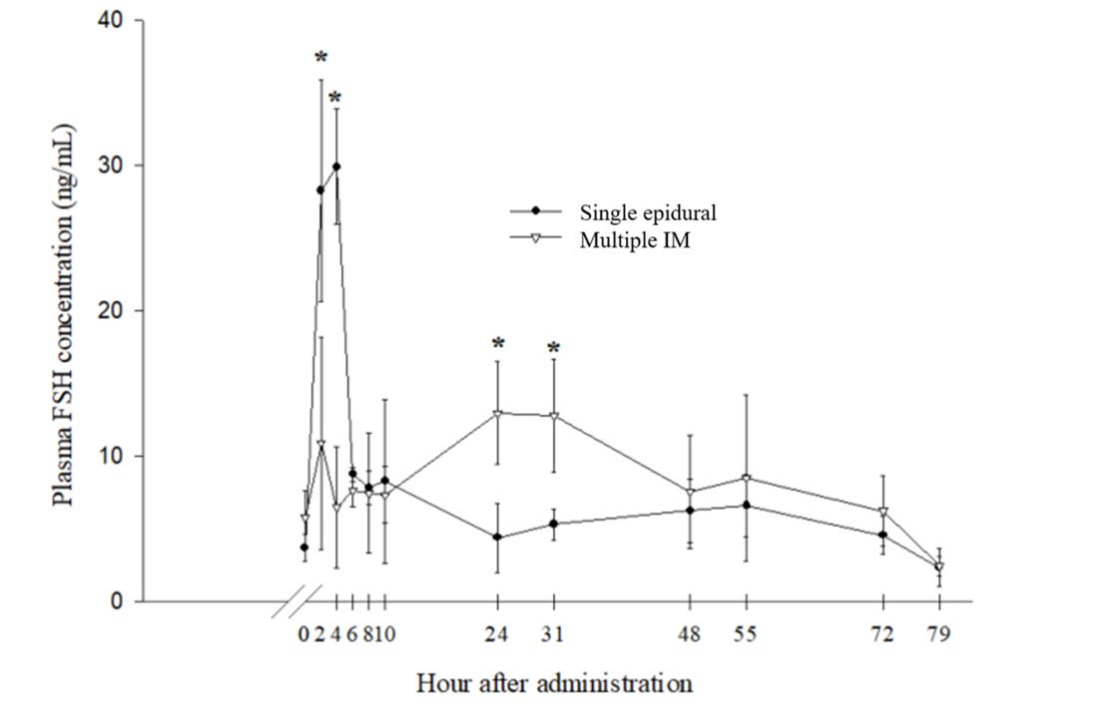
Mean±SE of plasma FSH in Thai-Holstein Friesian crosses breed treated with EI group and control group.

The results of efficacy of two treatment superstimulation protocols on FSH analyses are shown in Figure 2. The plasma concentration of FSH in the control group increased within 2 hours after FSH administration, and was relatively constant until 10 hours, being continuously maintained in the circulation until 72 hours. At 79 hours after the first injection, the levels of FSH decreased to a lower level compared with 0 hours before FSH administration. For the EI group, the plasma concentration of FSH was dramatically increased within 2 hours and reached a peak for about two hours before decreasing sharply thereafter (*P* < 0.01). After 10 hours of administration, the FSH levels in the EI groups decreased to a lesser level before FSH administration at 0 h. The level of FSH in the EI group was lower than in the control group at all times, but especially at 24 and 31 hours, which were significantly lower compared with the control (*P* < 0.05).

The results of ovarian response are presented in Table 2. According to the number of large follicles in the groups (Table 1), there were more in the control groups than in the EI group (*P*< 0.05); the administration of FSH by epidural injection resulted in a significantly lower number of CLs (15.3 ± 1.0 vs. 8.5 ± 1.3; *P* < 0.05), while the number of unovulated follicles was not different (*P* > 0.05). The maximum sizes of CLs were greater in the control group (24.1 ± 1.1 vs. 19.6 ± 1.3; *P* < 0.05). However, the maximum sizes of the unovulated follicles were significantly greater in the EI group compared with the control group (24.6 ± 2.7 vs. 14.6 ± 2.3; *P* < 0.05). The ovulation rate was not significant (*P* > 0.05). Moreover, the EI group showed an increase in ovarian cysts in cows (*P* < 0.01).

**Table 2:** The ovarian responses between cows administrated FSH by twice daily intramuscular injection or single epidural injection of 400 mg FSH in Thai-Holstein Friesian (Mean ± SE).

**Group**	**Corpus lutea (n)^1^**	**Maximum of CLs1**	**Unovulated follicles (n)2**	**Maximum of unovulated follicles(mm.)^2^**	**ovulation rate(%)3**	**No. of ovarian cyst in cow (n)**
Control	15.3±1.0^a^	24.1±1.1^a^	1.5±0.5^a^	14.6±2.3^a^	83.3±4.3^a^	1(8.3%)a
(*n=*12)						
EI	8.5±1.3^b^	19.6±1.3^b^	2.0±0.5^a^	24.6±2.7^b^	75.2±5.4^a^	6(50.0%)b
(*n=*12)						
*P*-values	0.0082	0.0353	0.5714	0.0456	0.2996	0.0047

^a,b^ For treatment effect, means within column are significantly different (P<0.05) by Duncan’s multiple range test (DMRT). Control: FSH 400 mg continuous intramuscular injection twice a day for 4 days. EI: FSH 400 mg single epidural injection. ^1^number/size of corpus luteal on flushing day; ^2^number/size of large follicles with a diameter > 9 mm. on flushing day; ^3^percent of ovulation rate (number of CLs / CLs and unovulated follicles X 100). SE is standard error of the sample mean calculated from $\frac{SD}{\sqrt{n}}$ when SD is standard deviation of sample size and $\sqrt{n}$ is square root of sample size.

The results of ova/embryo collection from the two superstimulation protocols are presented in Table 3. The numbers of ova/embryos recovered did not differ between groups (8.6 ± 1.4 and 4.9 ± 2.0; *P* > 0.05). However, the percentage of fertilized ova was significantly higher in the control group (74.4 and 40.8%; *P* < 0.05). Besides, the embryo quality was different between groups. The embryo quality in terms of transferable and freezable embryos was better in the control group than in the EI group (*P* < 0.05).

**Table 3:** The superovulatory response as determined by ova/embryo collected from cows administrated FSH by twice daily intramuscular injection or single epidural injection of 400 mg FSH in Thai-Holstein Friesian (Mean ± SE).

	**Total ova/embryo recovered (n)**	**Fertilized** **ova ^1^(%)**	**Transferable embryos1 (%)**	**Freezable embryo ^1^ (%)**
Control	8.6+1.4^a^	74.4^a^	66.3a	49.0^a^
EI	4.9+2.0^a^	40.8^b^	22.4b	14.3^b^
*P*-value	0.2667	0.0146	0.0397	0.0307

^a,b^ For treatment effect, means within column are significantly different (*P* < 0.05) by Duncan’s multiple range test (DMRT). Control: FSH 400 mg continuous intramuscular injection twice per day for 4 days. EI: FSH 400 mg single epidural injection. ^1^percent of quality embryo (number of quality /ova or embryo X 100). SE is standard error of the sample mean calculated from $\frac{SD}{\sqrt{n}}$ when SD is standard deviation of sample size and $\sqrt{n}$ is square root of sample size.

**Figure 3:**
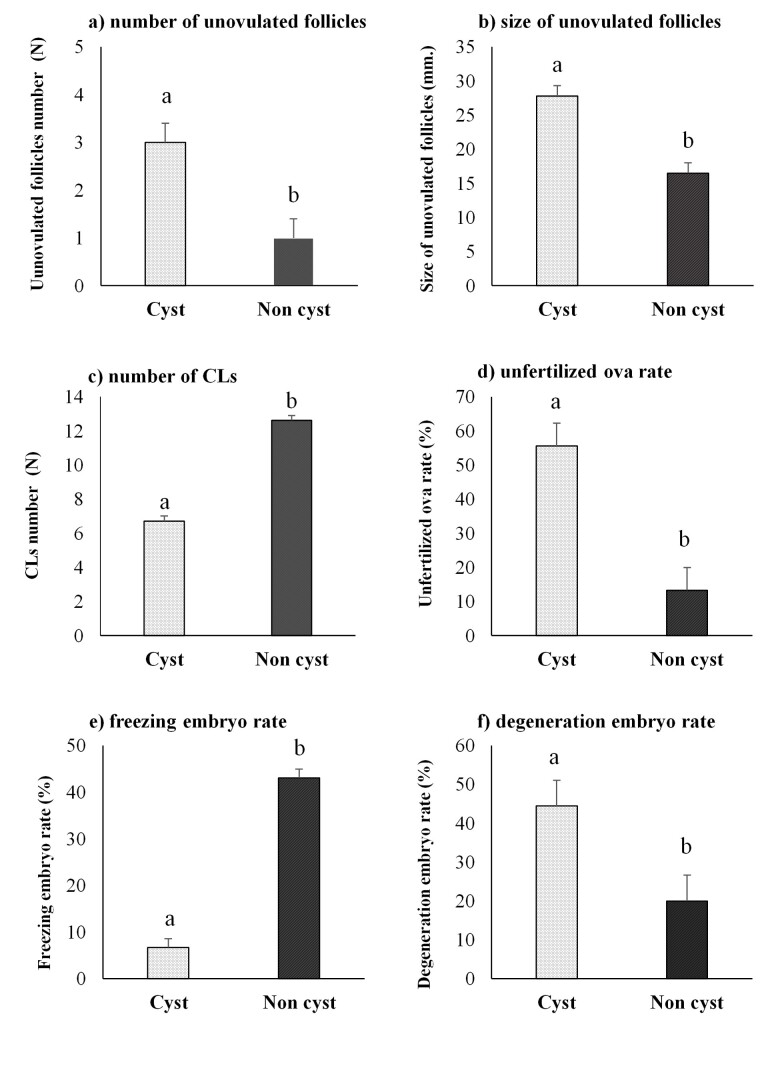
The ovarian responses and embryo quality in cyst and non-cyst cows (n=6 per group) administered FSH by a single epidural injection of 400 mg FSH in Thai-Holstein Friesian animals (Mean ±SE). ^a, b^ *P* < 0.05.

According to the ovarian responses (Table 2), the number of follicular cysts in the EI group was 50% (2/4 cows in non-response group and 4/8 cows in response group). Thus, the treatment cows were separated into cyst (n=6) and non-cyst (n=6) groups to compare the ovarian responses and embryo quality (Figure 3). In the cyst group, the numbers, and sizes of unovulated follicles were higher (*P* < 0.05), while the numbers of CLs were lower (*P* < 0.01) compared with the non-cyst group. In addition, the percentages of unfertilized ova and degenerate embryos were greater (*P* < 0.05), while there were fewer freezable embryos (*P* < 0.05) in the cyst group.

## Discussion

Superstimulation is apparently reported as the efficacy of tools to increase the number of high genetic animals and is widely used in many countries. However, the conventional method of ovarian superstimulation requires the multiple injection of gonadotropins, which is time-consuming and may also be stressful for cows. Many previous studies proposed solving this problem by administering FSH dissolved in various agents that provide a slow release, but those diluting agents mainly make the solution viscous and difficult to prepare well with FSH. A single epidural injection of FSH is therefore represented as an alternative method to induce follicular development. The present study revealed that total follicular responses during oestrus were not different between treatments; however, the large follicles and corpus luteum were lower (*P* < 0.01) in EI. The plasma concentration of FSH in EI was dramatically increased within 2 hours before decreasing sharply thereafter (*P* < 0.01) and was not maintained above baseline after 10 hours of administration. The embryo quality in terms of transferable and freezable embryos in control was better than in the EI group (*P* < 0.05). Interestingly, the number of ovulation cysts in the EI group was 50%. The ovarian responses and embryo quality in the cows with cysts were worse compared with non-cyst groups (*P* < 0.05).

Even though the total follicular responses during oestrus were not different between treatments, the large follicles (>9 mm) were lower (*P* < 0.01) while medium follicle sizes (5-8 mm) were higher (*P* < 0.05) in the EI group compared with control group. The difference in follicle size development was in accordance with the corpus luteum (fewer CLs in the EI group). The ovulation number followed the superstimulation treatment depending on the number of follicles, as this responds to treatment with proliferation, differentiation, and ovulation in a period of 120 hours (Kanitz et al., 2002). This speculates that the FSH concentration in a single epidural injection might not be sufficient to induce superstimulation, which is subsequently associated with anovulation in the Thai-Holstein crossbred. However, the ovulation rates were not different between groups which might be due to GnRH administration at 12 hours before the time of insemination in the present protocol to promote ovulation (Chankitisakul et al., 2017).

A continuously high level of gonadotropins, especially FSH, is necessary to stimulate the follicle development from the preantral to preovulatory stage (Gougeon, 1996; Picton et al., 2008). It is important to clarify whether decreasing follicle development in EI group resulted from low levels of circulating FSH with a single epidural injection. Therefore, FSH plasma concentrations were evaluated to confirm the activity. We found that the levels of plasma FSH in the EI group was dramatically increased within 2 hours and maintained for around 2 hours before decreasing sharply (*P* < 0.01). Besides, those could not maintain above baseline after 10 hours of administration. A drastic increase and then a sudden decline in plasma concentration of FSH was reported once by single injecting of FSH subcutaneously dissolved in saline and resulted in failure of follicular development and ovulation (Takedomi et al., 1995). Those were similar to our outcomes. In contrast with the EI group, the FSH level in the control group was continuously maintained in the circulation until 72 hours. This finding was in accordance with previous reports of circulating FSH levels that could induce follicular growth (Bó et al., 2018). Therefore, we inferred that a single injection of FSH in the epidural space is not appropriate in dairy cows, at least in our study. The epidural fats could not contribute to the slow movement of FSH into the peripheral circulation, as also seen successfully in beef cattle (Sakaguchi et al., 2018). However, satisfying results in beef cattle with the single subcutaneous administration of FSH which was non-repeatable in dairy cows that had less adipose tissue, were reported previously (Hockley et al., 1992; Lovie et al., 1994).

Taken together, the percentages of embryo quality in terms of either transfer or freezing embryos showed significant differences between epidural injection and IM injection. This might be explained by the follicle and corpus luteum sizes in this study, which were larger in the latter group (*P* < 0.05). The effect of follicle size at oestrus influences the corpus luteum size, subsequently increasing progesterone level, which correlated with the enlargement in CL size (Vasconcelos et al., 2001; Busch et al., 2008; Pandey et al., 2018). The CL is a transient reproductive gland that produces progesterone, and is responsible for progesterone secretion, which is required for the establishment and maintenance of pregnancy (Schams and Berisha, 2004). Therefore, the higher percentages of transferable and freezing embryos in the control group indicated that a greater follicle size revealed greater CL diameters, consequently leading to better embryo quality.

Interestingly, in the present study, the sizes of unovulated follicles at the time of embryo flushing were larger in the EI group (*P* < 0.05). It seems plausible that those unovulated follicles developed into follicular cysts in a single epidural injection protocol, as the secondary follicles that fail to ovulate could form follicular cysts. If the follicle does not rupture, it instead becomes a cyst (Cole and Kramer, 2016). Even though GnRH was administered to induce ovulation in the present study, ovulation of the dominant follicle depends on status in terms of the growth, static nature, or regression of those dominant follicles at the time of GnRH injection (Twagiramungu et al., 1992; Silcox et al., 1993). This evidence was rarely mentioned, however, in accordance with the higher medium follicle sizes during oestrus in the EI group; we therefore speculated that the administration of GnRH could not induce ovulation in any follicle where improper status resulted in anovulatory follicles and subsequently those developed into cysts.

The impact of cysts was found in the ovarian follicular response and embryo quality (Figure3). The numbers and sizes of unovulated follicles were greater, while the numbers of CLs were lower in the cyst group (*P* < 0.05). Besides, ovarian cysts in a single epidural injection have disadvantages over fertilized ova and embryo quality. This could be explained by high estrogen in the follicular fluid of ovarian cysts interfering with the embryo and retard fetal and placental growth in mammals (Bartholomeusz et al., 1999).

## Conclusion

In conclusion, these results indicate that the epidural administration of FSH was improper for alternative techniques in the Thai-Holstein crossbreed. The epidural injection of FSH could not maintain sufficient FSH levels and subsequently affected a decrease in superovulatory response and increase in poor embryo quality. Also, the incidence of ovarian follicular cysts is higher in the EI group. Further studies, such as examination of dose of FSH for injection in epidural space and difference in effect of EI method among breeds, should be performed to determine the efficacy of EI methods for induction of superovulation in cattle.
